# Special Issue: Coevolution of Hosts and Their Microbiome

**DOI:** 10.3390/genes9110549

**Published:** 2018-11-13

**Authors:** Morten T. Limborg, Philipp Heeb

**Affiliations:** 1 Natural History Museum of Denmark, University of Copenhagen, 1165 Copenhagen, Denmark; 2 Laboratoire Évolution et Diversité Biologique (EDB), UMR 5174 Centre National de la Recherche Scientifique (CNRS), Université Paul Sabatier, 31330 Toulouse, France

The evolution of life-history traits in plants and animals has taken place in the midst of complex microbial communities. Biology is undergoing a fundamental reshaping where the phenotypic expression of the individuals’ traits need to be considered as the combined expression of the host and its associated microbial genomes, defined as forming the “holobiont” (i.e., the host and its microbiota) [1,2]. This new concept has wide ranging implications and has led to the realization that multicellular organisms coevolve with their microbial symbionts [3]. Although host–microbe interactions can be understood and explained through ecological processes, much less is known about the significance of evolutionary and eco-evolutionary processes in shaping the holobionts.

Given the complex structure of the microbiome, understanding the specific roles, adaptability, and functions provided by the microbiome to its host is emerging as an exciting new scientific frontier [4]. The close contact between the microbiota and their host means that the microbiota can shape a variety of effects ranging from physiological trade-offs to behavioral and cognitive traits [5,6].

In this special issue we present four papers that provide empirical examples and review recent developments in molecular and statistical techniques to discuss theoretical concepts and empirical evidence on the potential role of the microbiota in shaping holobiont evolution. Although the papers in this issue all focus on animal systems, we stress that the importance of host–microbiome interactions can be readily transferred to any other multicellular organism including plants [7].

An exciting area of host–microbiome research relates to the function played by microorganisms in olfactory communication among their host organisms and its evolutionary implications. While this remains a little studied topic, Maraci et al. [8] provides an exhaustive review of this emerging field with specific focus on (i) key body regions serving as microbial hotspots for olfactory signaling, (ii) sources of microbes, (iii) functional aspects in social communication, and (iv) evolutionary implications. They advocate the use of birds as model systems due to the fact that the effects of the microbiome are establishing the importance of olfactory communication in birds coupled with uropygial gland secretions used to preen and protect feathers. Indeed, the uropygial gland and its interactions with the feather microbiota distinguish birds from other vertebrates for which mainly the gut microbiota has been considered. They also draw on results from the literature on other taxa including humans and insects, illustrating the relevance of studying host–microbiome interactions to better understand the use of smells in communication.

Indeed, the key role played by the uropygial gland microbiome in birds is further scrutinized in the study by Rodriguez-Ruano et al. [9]. The authors apply multiple methods to survey the microbiota profile in the uropygial gland among sexes, life stages, and reproductive status of the hoopoe (*Upupa epops*). All these parameters turned out to be relevant in that a denser and more diverse microbiota were observed in the darker secretions from the uropygial gland that are a characteristic of nesting individuals. Interestingly, this dark secretion was also characterized by having a higher concentration of known antibiotic-producing bacteria such as *Vagococcus* and *Parascardovia* strains. As such, the study exemplifies the multifunctional role of the uropygial gland microbiota by also serving important anti-microbial functions. The authors hypothesize that this function is an adaptation to the higher infection risk of nestling birds and thus adds to our understanding of the important role of host-microbiome interactions in boosting anti-bacterial responses against pathogenic infections. The hoopoe is a particularly relevant biological model since their preen glands are colonized by specific bacterial strains, a pattern that seems to be present in a growing number of species.

Another outstanding question within this nascent field is to understand the relative contributions of diet versus host genotypic background in shaping the gut microbiome composition. In this issue, Ruiz-Rodriguez et al. [10] address this question in an elegant study design using the brood-parasitizing great spotted cuckoo (*Clamator glandarius*) as a model system. They compare gut microbiome profiles among adult cuckoos, nestling cuckoos, and the parasitized magpie (*Pica pica*) nestlings. Since vertical transmission of microorganisms is considered to be very limited from parent to offspring cuckoos because the eggs hatch in the parasitized species nest, any similarities between nestling cuckoos and their parents can be explained by host genetic effects while similarities with the magpie nestlings can be explained by the diet (environment). Their results show that both host genotypic background as well as diet determines gut microbiomes in nestling cuckoos. While the importance of diet in shaping the gut microbiota composition seems less surprising, it is interesting that they observe a clear effect of host genetic background. Indeed, this tells us that evolutionary processes affect the gut microbiome over generations.

In their review paper, Pasquaretta et al. [11] take as a given the feedback interactions between diet and gut microbiota composition and propose to use models of nutritional ecology (nutritional geometry) to explore the complex interactions between diet selection, microbiota composition, and host fitness. Since both host and microbiota aim to reach their own intake target (theoretical amount and ratio of different food types) it becomes relevant to examine the relative roles of the microbiota and its host in achieving the holobiont intake target through different nutritional strategies mirroring the action of the specific components. They propose that nutritional constraints imposed by the microbiota on the host intake target can lead to deviations away from the host optimum. Pasquaretta et al. also discuss how these individual level interactions can drive group level effects by shaping a wide range of social behaviors and social structures in animal groups. The authors conclude that nutritional geometry provides a powerful framework to examine the interplay between the host and its microbiota, shaping the holobiont nutrition both theoretically and experimentally. The elegance of this approach is that it can be applied to all animals. 

Together, the studies and reviews presented in this issue reflect the broader scope of research fields and applications where we foresee exciting and speedy developments of research on host–microbiome coevolution. For example, individual case studies on host–microbiome interactions contribute to a deeper understanding and development of the holobiont concept in evolutionary biology [12,13]. This will lead to wider impacts beyond the field of evolutionary biology including a more efficient use of microbiome research in food production [14] and human disease studies [15]. It is our hope that this special issue will help steer this development.
